# Mental Health Outcomes in Healthcare Workers in COVID-19 and Non-COVID-19 Care Units: A Cross-Sectional Survey in Belgium

**DOI:** 10.3389/fpsyg.2020.612241

**Published:** 2021-01-05

**Authors:** Julien Tiete, Magda Guatteri, Audrey Lachaux, Araxie Matossian, Jean-Michel Hougardy, Gwenolé Loas, Marianne Rotsaert

**Affiliations:** ^1^Service de Psychologie, CUB-ULB Hôpital Erasme, Brussels, Belgium; ^2^Faculté des Sciences Psychologiques et de l’Éducation, Université libre de Bruxelles, Brussels, Belgium; ^3^Service de Psychologie, Hôpital de Jolimont, La Louvière, Belgium; ^4^Service de Néphrologie, CUB-ULB Hôpital Erasme, Brussels, Belgium; ^5^Service de Psychiatrie, CUB-ULB Hôpital Erasme, Brussels, Belgium

**Keywords:** COVID-19, healthcare workers, distress, burnout, Health Psychology

## Abstract

**Background:**

The literature shows the negative psychological impact of the coronavirus disease 2019 (COVID-19) outbreak on frontline healthcare workers. However, few are known about the mental health of physicians and nurses working in general hospitals during the outbreak, caring for patients with COVID-19 or not.

**Objectives:**

This survey assessed differences in mental health in physicians and nurses working in COVID-19 or non-COVID-19 medical care units.

**Design:**

A cross-sectional mixed-mode survey was used to assess burnout, insomnia, depression, anxiety, and stress.

**Setting:**

A total of 1,244 physicians and nurses from five general hospitals in Belgium, working in COVID-19 care units (CCU), non-COVID-19 care units (NCCU), or both (CCU + NCCU) were informed of the study.

**Participants:**

Six hundred forty-seven healthcare workers participated in the survey (response rate = 52%).

**Measurements:**

Validated instruments were used to assess the outcomes: the *PFI* (burnout/professional fulfillment), the *ISI* (insomnia), and the *DASS-21* (depression, anxiety, and stress).

**Results:**

Results showed high prevalence of burnout, insomnia, depression, and anxiety among participants. After adjusting for confounders, multivariate analysis of variance showed no differences between CCU, NCCU, and CCU + NCCU workers. Univariate general linear models showed higher level of burnout, insomnia, and anxiety among nurses in comparison to physicians. Being a nurse, young, isolated, with an increased workload were risk factors for worse mental health outcomes.

**Limitations:**

The mental health of the tested sample, before the outbreak, is unknown. Moreover, this cross-sectional design provides no information on the evolution of the mental health outcomes over time.

**Conclusion:**

Directly caring for patients with COVID-19 is not associated with worse mental health outcomes among healthcare workers in general hospitals. High prevalence of burnout, insomnia, depression, and anxiety among physicians and nurses requires special attention, and specific interventions need to be implemented.

**Protocol Registration:**

ClinicalTrials.gov, identifier NCT04344145.

## Introduction

The coronavirus disease 2019 (COVID-19) is a respiratory infectious disease caused by severe acute respiratory syndrome coronavirus 2 (SARS-CoV-2), first reported in China in December 2019, which has spread globally, resulting in a worldwide pandemic (Li et al., 2020). In Belgium, 6,09,211 confirmed cases of COVID-19 were reported for the period from March 1st to December 15th, 2020, with a total of 19,055 hospitalization and 18,054 deaths (Sciensano, 2020). Relative to its population, Belgium has the highest death per inhabitant ratio (Johns Hopkins Coronavirus Resource Center, 2020).

Given the contagion, severity, and mortality characteristics of the disease, the COVID-19 outbreak has led to clinical, organizational, and technical challenges (Cook et al., 2020; Meo et al., 2020; Wu Y.C. et al., 2020). Recent studies reported the negative psychological impact of the COVID-19 outbreak on healthcare workers (Kang et al., 2020; Lai et al., 2020; Lu et al., 2020; Tan et al., 2020). One study reported that 50.4% of healthcare workers presented symptoms of depression, 44.6% anxiety, 34.0% insomnia, and 71.5% distress (Lai et al., 2020). These impacts were particularly important among nurses, who tend to report more severe symptoms of insomnia and emotional distress (Lai et al., 2020). Most importantly, studies also reported that working on the frontline is a risk factor for worse mental health outcomes (Lai et al., 2020; Lu et al., 2020). Previous studies have also reported negative psychological impacts of the SARS outbreak (Maunder et al., 2003, 2006; Bai et al., 2004; Lee et al., 2007; Lancee et al., 2008; Lung et al., 2009) and the H1N1 influenza (Goulia et al., 2010; Matsuishi et al., 2012). Healthcare workers showed high levels of depression, anxiety, and stress up to 1 year post SARS outbreak (Lee et al., 2007), and higher levels of psychological distress and posttraumatic stress were reported among those who directly cared for patients with SARS (Maunder et al., 2006). In addition, uncertainty, fear of the unknown, fear of contagion, and of being a risk of infection to their colleagues, families, and friends were frequently reported by workers (Maunder et al., 2003). Similar consequences were reported during H1N1 influenza outbreak, with evidence of higher levels of anxiety among nurses, young workers and those directly treating and caring for patients with H1N1 (Matsuishi et al., 2012).

Just like the SARS and H1N1 outbreaks, the management of COVID-19 thus implies high-risk work environment in terms of stress, contagion, or increased workload for healthcare workers. However, it is a well-known fact that occupational chronic stress with emotionally intense work load/demands in a context where resources are inadequate can result in burnout (Schaufeli et al., 2009). Burnout is a psychological syndrome characterized by an emotional exhaustion, depersonalization, and a sense of lack of personal fulfillment (Schaufeli et al., 2009). Burnout negatively affects healthcare workers and, by extension, the entire healthcare system and its patients. Several studies report that burnout is associated with lower work satisfaction, more substance abuse behaviors, depression, relational concerns, suicidal thoughts, and suicide (van der Heijden et al., 2008; Wurm et al., 2016). Within healthcare organizations, such as hospitals, burnout is associated with reduced productivity and higher job turnover (Shanafelt et al., 2016). Burnout also reduces quality and security of patient care and patients’ satisfaction (West et al., 2006, 2009; Fahrenkopf et al., 2008). The key risk factors for burnout are imbalance between mastered skills and required skills in medical situations, excessive workload, prolonged work stress, and a perceived lack of control (Linzer et al., 2001). A recent study reported lower rate of burnout among frontline COVID-19 healthcare workers (13%) than among non-COVID-19 workers (39%) (Wu Y. et al., 2020). Authors interpreted these results to mean that it is likely that frontline workers have a greater sense of control, specifically in their work environment (Wu Y. et al., 2020). In 2017, a national Belgian study reported that 5% of physicians and 6% of nurses presented a significant level of burnout, including dimensions of emotional exhaustion, depersonalization, and a lack of personal fulfillment (Vandenbroeck et al., 2017). Moreover, 12% of physicians and 17% of nurses are at risk of burnout, including at least two of these dimensions.

Healthcare workers’ burnout and mental health during the COVID-19 outbreak are thus a critical public health issue and have to be studied more precisely. Several studies have been published on the psychological impact of the COVID-19 outbreak on healthcare workers. However, few studies have assessed potential differences in mental health among COVID-19 healthcare workers and non-COVID-19 healthcare workers. When they did, they generally compared groups of workers at high or low risk of being in contact with patients with COVID-19 (Kang et al., 2020; Lu et al., 2020). Only one has formally compared frontline workers to workers in usual care units and only for burnout (Wu Y. et al., 2020). Moreover, most of these studies have been conducted in Asia, and their results could be associated with cultural differences in healthcare system and how healthcare workers react to crises. To address these gaps in comparable data in healthcare workers’ mental health, this current study aimed to evaluate potential differences in burnout and mental health outcomes among COVID-19 healthcare workers and non-COVID-19 healthcare workers in five general hospitals in Belgium. We hypothesized that prevalence and severity of burnout, insomnia, depression, anxiety, and stress will be greater among healthcare workers in COVID-19 care units (CCU) in comparison to those in non-COVID-19 care units (NCCU).

## Materials and Methods

### Design

The study was a cross-sectional mixed-mode survey on healthcare workers in five general hospitals in Belgium during the COVID-19 outbreak. Recruitment was active from April 17th to May 25th, 2020.

All study procedures were approved by the Ethics Committee of Erasme Hospital (P2020/221) and Jolimont-Lobbes Hospital. Study protocol has been registered at ClinicalTrials.gov (NCT04344145).

### Sample

At the start of the COVID-19 outbreak in Belgium, the five participating general hospitals reorganized their medical care units with workers involved and working either solely in CCU, solely in NCCU or in both COVID-19 care units and NCCU (CCU + NCCU). CCU were composed of intensive and non-intensive COVID-19 care units. NCCU were composed of intensive and non-intensive NCCU. Inclusion criteria included the ability to speak and read French and being professionally active in CCU or NCCU (or both). Workers who were professionally inactive for 3 weeks or more were excluded. Physicians and nurses who met the inclusion criteria were informed by email about the study. The sample size was calculated for a 95% CI, with a tolerated margin error of 5% and an expected prevalence of emotional distress of 50%, based on a recent study (Lai et al., 2020), using the formula *N* = *Z*_α_^2^*P*(1− *P*)/*d*^2^ in which *Z*_α_^2^ = 1.96, *P* = 0.5, and *d*^2^ = 0.0025. To allow subgroup analyses, this number was then amplified by 50%; to set the goal, at least 576 surveys were needed to be completed. This number was then doubled to allow for an estimated response rate of 50%. Thus, 1,244 healthcare workers were invited to participate in the study.

### Outcomes Measurements

To test our hypothesis, a number of validated measurements were used. First, sociodemographic characteristics were gathered using an 11-item questionnaire. Items provided information on age, gender, marital status, education, psychiatric history, and perceived social support. Perceived social support was assessed with a single item on a 3-point Likert scale (“Poor,” “Average,” “Good”): “How do you define the social support you receive?”

Professional information was also gathered, using a 10-item questionnaire. Items provided information on occupation, working position, job status, years of experience, and workload.

Professional fulfillment and burnout were measured using the *Stanford Professional Fulfillment Index* (*PFI*), validated on hospital physicians (Trockel et al., 2018). This is a 16-item scale, divided into 2 subscales: professional fulfillment (6 items) and burnout, including professional exhaustion (4 items) and interpersonal disengagement (6 items). The PFI uses a 5-point Likert scale. Response options range from “not at all true” to “completely true” (0–4 score range) for the professional fulfillment subscale and from “not at all” to “extremely” (0–4 score range) for the burnout subscale, based on a 2-week recall period. Subscale scores are provided by the mean of all subscale items (professional fulfillment, 0–4; burnout, 0–4). Cutoff scores are set at ≥3 for the fulfillment subscale (significant professional fulfillment) and at ≥1.33 for the burnout subscale (significant burnout).

Emotional distress was measured with the *Depression, Anxiety, and Stress Scale* (*DASS*), 21-item version (Lovibond and Lovibond, 1995; Antony et al., 1998). The DASS is divided into three 7-item subscales: depression, anxiety, and stress. The DASS has a 4-point Likert scale. Responses options range from “Never” to “Almost always” (0–3 score range), based on a 1-week recall period. Subscales scores are given by the sum of all subscale items. Cutoffs can be used for depression (normal, 0–9; mild, 10–13; moderate, 14–20; severe, 21–27; and extremely severe, ≥28), anxiety (normal, 0–7; mild, 8–9; moderate, 10–14; severe, 15–19; and extremely severe, ≥20) and stress severity (normal, 0–14; mild, 15–18; moderate, 19–25; severe, 26–33; and extremely severe, ≥34).

Finally, sleep disturbance was measured with the *Insomnia Severity Index* (*ISI*) (Morin, 1993). This is a 7-item scale that has a 5-point Likert scale (0–4 score range). Response options range from “none” to “very severe” for items 1–3, from “very satisfied” to “very dissatisfied” for item 4 and from “none” to “very much” for items 5–7, based on a 4-week recall period. The ISI provides a total score by summing all items scores. Cut-off scores are set for no clinically significant insomnia (0–7), subthreshold insomnia (8–14), moderate clinical insomnia (15–21), and severe clinical insomnia (22–28).

### Data Collection

Information about the study was first sent out by email. This was followed by a reminder 2 weeks later. Participants could fill out the survey either electronically or on paper. LimeSurvey^TM^ was used for the electronic version. Paper versions were available in every medical care unit if participants were not able to fill out the electronic version. Both versions were anonymous. The principal and local investigators checked daily for newly completed surveys in medical care units. Electronic data were stored on the associated LimeSurvey^TM^ server of the Université libre de Bruxelles. Paper data were stored in a secured place in each participating hospital.

### Statistical Analyses

Statistical analysis consisted first in a comparative analysis of participants’ sociodemographic and professional characteristics using Chi-squared test for each categorical variable. Second descriptive analyses were performed on severity categories for professional fulfillment, burnout, insomnia, depression, anxiety, and stress. Differences between groups regarding severity levels were tested using χ^2^. Multivariate general linear models (GLMs) were used to test main effects of medical care unit type (group effect), occupation type (occupation effect), and to test a group × occupation interaction effect on fulfillment, burnout, insomnia, and mental health scores. GLM were adjusted for age, gender, marital status, workload, and job status. Multivariable logistic regression analyses were performed to estimate risk factors for burnout, clinically significant insomnia and severe to extremely severe depression, anxiety, and stress. These were presented as odds ratios, with 95% confidence intervals, adjusting for confounders. All tests were two-tailed, and alpha was set at 0.05. All analyses were performed using IBM SPSS v26.

## Results

### Sociodemographic and Professional Characteristics

A total of 647 healthcare workers participated (response rate = 52%). All sociodemographic and professional characteristics are presented in Table 1. The majority of the participants (72.3%) were nurses. More than half had 10 years or more work experience and an undergraduate level of education or less. Half (50.4%) of the participants worked in CCU, while the other half worked in NCCU (38.2%) and in both CCU and NCCU (11.4%). Half of them reported working 40 h or more per week. One out of three participants reported an increase in their workload since the beginning of the COVID-19 outbreak. Most participants were women, were married or in relationship, and were aged 31 years or more. A Chi-squared test highlighted that participants in the NCCU group were older than participants in the CCU group or participants in the CCU + NCCU group, χ^2^ (4, *N* = 647) = 28.8, *p* < 0.001. A Chi-squared test also highlighted that participants in the CCU group reported an increase in their workload more frequently than in the NCCU group or in CCU + NCCU group, χ^2^ (1, *N* = 647) = 46.8, *p* < 0.001.

**TABLE 1:** Demographic and professional characteristics of participants.

	**No. (%)**			
		**Working position**		
**Characteristics**	**Total**	**COVID-19 care unit (CCU)**	**Non-COVID-19 care unit (NCCU)**	**Both (CCU + NCCU)**
Overall	647 (100)	326 (50.4)	247 (38.2)	74 (11.4)
**Gender**				
Male	140 (21.6)	81 (24.8)	45 (18.2)	14 (18.9)
Female	507 (78.4)	245 (75.2)	202 (81.8)	60 (81.1)
**Age**				
20–25 years	41 (6.3)	23 (7.1)	13 (5.3)	5 (6.8)
26–30 years	108 (16.7)	63 (19.3)	35 (14.2)	10 (13.5)
31–40 years	179 (27.7)	99 (30.4)	55 (22.3)	25 (33.8)
41–50 years	157 (24.3)	85 (26.1)	53 (21.5)	19 (25.7)
>50 years	162(25.0)	56 (17.2)	91 (36.8)	15 (20.3)
**Marital status**				
Single	126 (19.5)	64 (19.6)	47 (19.0)	15 (20.3)
Married	521 (80.5)	262 (80.4)	200 (81.0)	59 (79.7)
**Education**				
≤Undergraduate	402(62.1)	210 (64.4)	149 (60.3)	43 (58.1)
Graduate	190 (29.4)	91 (27.9)	75 (30.4)	24 (32.4)
Postgraduate	55 (8.5)	25 (7.7)	23 (9.3)	7 (9.5)
**Occupation**				
Physician	179 (27.7)	87 (26.7)	69 (27.9)	23 (31.1)
Nurse	468 (72.3)	239 (73.3)	178 (72.1)	51 (68.9)
**Experience**				
0–5 years	140 (21.7)	83 (25.5)	41 (16.6)	16 (21.7)
5–10 years	112 (17.3)	62 (19.0)	33 (13.4)	17 (23.0)
10–20 years	162 (25.0)	83 (25.5)	60 (24.3)	19 (25.7)
>20 years	233(36.0)	98 (30.1)	113 (45.7)	22 (29.7)
**Weekly workload**				
20–40 h	322 (49.7)	131 (40.2)	153 (61.9)	38 (51.4)
40–50 h	224 (34.6)	128 (39.3)	70 (28.3)	26 (35.1)
>50 h	101 (15.7)	67 (20.6)	24 (9.7)	10 (13.5)
**Increased workload**				
Yes	214 (33.1)	150 (46.0)	48 (18.6)	18 (24.3)
No	433 (66.9)	176 (54.0)	201 (81.4)	56 (75.7)

### Severity of Symptoms

Nearly half of the participants reported a significant level of burnout, and only one out of three participants reported being professionally fulfilled. Two out of the three participants had symptoms of insomnia. Nurses and CCU workers reported more severe symptoms of insomnia than physicians and NCCU and CCU + NCCU workers, respectively (e.g., 36.1% of nurses reported moderate or severe symptoms of insomnia vs. 24.1% of physicians, *p* = 0.002; 36.8% of CCU workers reported moderate or severe symptoms of insomnia vs. 28.8% of NCCU workers and 28.4% of NCCU + CCU workers, *p* = 0.042). Regardless of the care unit type, half of the participants had symptoms of depression and anxiety, and little less than half of the participants had symptoms of stress. More precisely, one out of four participants (28.8%) had moderate to extremely severe symptoms of depression, two out of five participants (41.8%) had moderate to extremely severe symptoms of anxiety, and one out of four participants had moderate to extremely severe symptoms of stress (25.1%). Nurses and female participants reported symptoms of anxiety more frequently than physicians and male participants, respectively (i.e., 63.2% of nurses had symptoms vs. 23.5% of physicians, *p* < 0.001; 57.4% of female participants had symptoms vs. 33.6% of male participants, *p* < 0.001) (Table 2).

**TABLE 2:** Severity categories of professional fulfillment, burnout, insomnia, and emotional distress measures by groups (*N* = 647).

	**No. (%)**										
		**Gender**			**Occupation**			**Working position**			
**Measurement**	**Total**	**Male**	**Female**	***p***	**Physician**	**Nurse**	***p***	**COVID-19 care unit (CCU)**	**Non-COVID-19 care unit (NCCU)**	**Both (CCU + NCCU)**	***p***
**PFI—professional fulfillment**											
Fulfilled	197 (30.4)	47 (33.6)	150 (29.6)	0.364	50 (27.9)	147 (31.4)	0.390	108 (33.1)	70 (28.3)	19 (25.7)	0.298
**PFI—burnout**											
Burnout	295 (45.6)	67 (47.9)	228 (45.0)	0.544	73 (40.8)	222 (47.4)	0.128	163 (50.0)	99 (40.1)	33 (44.6)	0.061
**ISI**											
Absence	202 (31.3)	55 (39.3)	147 (29.0)	0.103	74 (41.3)	128 (27.4)	0.002	83 (25.5)	92 (37.2)	27 (36.5)	0.042
Subthreshold	233 (36.0)	45 (32.1)	188 (37.1)	62 (34.6)	171 (36.5)	123 (37.7)	84 (34.0)	26 (35.1)
Moderate	169 (26.1)	34 (24.3)	135 (26.6)	37 (20.7)	132 (28.2)	98 (30.1)	57 (23.1)	14 (18.9)
Severe	43 (6.6)	6 (4.3)	37 (7.3)	6 (3.4)	37 (7.9)	22 (6.7)	14 (5.7)	7 (9.5)
**DASS-21—depression**											
Normal	302 (46.7)	67 (47.9)	235 (46.4)	0.776	86 (48.0)	216 (46.2)	0.817	151 (46.3)	113 (45.7)	38 (51.4)	0.981
Mild	159 (24.6)	38 (21.7)	121 (23.9)	41 (22.9)	118 (25.2)	79 (24.2)	62 (25.1)	18 (24.3)
Moderate	119 (18.4)	24 (17.1)	95 (18.7)	35 (19.6)	84 (17.9)	61 (18.7)	45 (18.2)	13 (17.6)
Severe	38 (5.9)	6 (4.3)	32 (6.3)	8 (4.5)	30 (6.4)	21 (6.4)	14 (5.7)	3 (4.1)
Extremely severe	29 (4.5)	5 (3.6)	24 (4.7)	9 (5.0)	20 (4.3)	14 (4.3)	13 (5.3)	2 (2.7)
**DASS-21—anxiety**											
Normal	309 (47.8)	93 (66.4)	216 (42.6)	<0.001	137 (76.5)	172 (36.8)	<0.001	146 (44.8)	122 (49.4)	41 (55.4)	0.170
Mild	67 (10.4)	10 (7.1)	57 (11.2)	9 (5.0)	58 (12.4)	41 (12.6)	19 (7.7)	7 (9.5)
Moderate	142 (21.9)	16 (11.4)	126 (24.9)	13 (7.3)	129 (27.6)	69 (21.2)	63 (25.5)	10 (13.5)
Severe	63 (9.7)	8 (5.7)	55 (10.8)	6 (3.4)	57 (12.2)	37 (11.3)	20 (8.1)	6 (8.1)
Extremely severe	66 (10.2)	13 (9.3)	53 (10.5)	14 (7.8)	52 (11.1)	33 (10.1)	23 (9.3)	10 (13.5)
**DASS-21—stress**											
Normal	386 (59.7)	97 (69.3)	289 (57.0)	0.098	112 (62.6)	274 (58.5)	0.379	182 (55.8)	156 (63.2)	47 (63.5)	0.382
Mild	99 (15.3)	14 (10.0)	85 (16.8)	23 (12.8)	76 (16.2)	54 (16.6)	35 (14.2)	10 (13.5)
Moderate	91 (14.1)	17 (12.1)	74 (14.6)	22 (12.3)	69 (14.7)	52 (16.0)	31 (12.6)	8 (10.8)
Severe	58 (9.0)	9 (6.4)	49 (9.4)	20 (11.2)	38 (8.1)	28 (8.6)	22 (8.9)	8 (10.8)
Extremely severe	13 (2.0)	3 (2.1)	10 (2.0)	2 (1.1)	11 (2.4)	10 (3.1)	3 (1.2)	1 (1.4)

*PFI, 16-item Stanford Professional Fulfillment Index; ISI, 7-item Insomnia Severity Index; DASS-21, 21-item Depression, Anxiety, and Stress Scale.*

### Multivariate and Univariate Analysis

All dependent variables (2-PFI subscales scores, 3-DASS-21 subscales scores, and ISI total score) were included in a multivariate GLM testing main effect for group, occupation, and a group × occupation interaction effect. Multivariate analysis indicates significant main effect for group [Pillai’s Trace = 0.04, *F*(12, 1,274) = 2.18, *p* = 0.017] and for occupation [Pillai’s Trace = 0.09, *F*(6,636) = 16.33, *p* < 0.001]. Group × occupation interaction effect is not significant. When adjusting for confounders, GLM multivariate analysis remains significant for occupation [Pillai’s Trace = 0.13, *F*(6,635) = 16.59, *p* < 0.001] but not for group, indicating that being a physician or a nurse is more predictive of the outcomes than working in CCU, NCCU, or both. After adjustment, GLM univariate analysis for occupation reported significant effect on burnout [*F*(1,645) = 13.57, *p* < 0.001], insomnia [*F*(1,645) = 23.04, *p* < 0.001], and anxiety [*F*(1,645) = 55.36, *p* < 0.001]. Adjusted GLM univariate analyses for each outcome are summarized in Table 3.

**TABLE 3:** Means, standard deviations, and adjusted* GLM univariates for professional fulfillment, burnout, insomnia, and emotional distress by groups (*N* = 647).

	**Mean (SD)**							
		**Occupation**			**Working position**			
**Measurement**	**Total**	**Physician**	**Nurse**	***p***	**COVID-19 are unit (CCU)**	**Non-COVID-19 care unit (NCCU)**	**Both (CCU + NCCU)**	***p***
**PFI**								
Fulfillment	2.5 (0.8)	2.4 (0.8)	2.5 (0.8)	0.055	2.5 (0.8)	2.5 (0.7)	2.4 (0.9)	0.863
Burnout	1.3 (0.7)	1.2 (0.7)	1.4 (0.7)	< 0.001	1.4 (0.7)	1.2 (0.8)	1.3 (0.7)	0.242
ISI	11.2 (6.6)	9.4 (6.3)	12.0 (6.5)	< 0.001	12.0 (6.4)	10.5 (6.6)	10.4 (7.1)	0.079
**DASS-21**								
Depression	10.8 (7.8)	10.5 (8.3)	10.9 (7.7)	0.150	10.9 (7.8)	11.1 (8.0)	9.8 (7.5)	0.248
Anxiety	8.9 (7.2)	5.8 (6.8)	10.2 (7.0)	< 0.001	9.2 (7.0)	8.8 (7.1)	8.6 (8.1)	0.844
Stress	14.1 (8.2)	13.6 (8.5)	14.3 (8.1)	0.058	14.8 (8.2)	13.6 (8.2)	13 (7.6)	0.335

PFI, 16-item Stanford Professional Fulfillment Index; ISI, 7-item Insomnia Severity Index; DASS-21, 21-item Depression, Anxiety, and Stress Scale. *For gender, age, job experience, and education.

### Risk Factors

After adjusting for confounders, logistic regression analysis indicated that an increase in workload is associated with burnout and severe to extremely severe symptoms of stress for all participants (e.g., stress among healthcare workers reporting increased workload: OR, 2.46; 95% CI, 1.46–4.14). It also showed that being a nurse and being young were associated with severe to extremely severe symptoms of anxiety (e.g., anxiety among 20–25 years: OR, 5.59; 95% CI, 1.48–21.21). Perceived poor social support is associated with burnout and severe to extremely severe symptoms of depression (i.e., depression among healthcare workers reporting poor social support: OR, 7.28; 95% CI, 2.62–20.23). Significant risk factors for the associated outcomes are summarized in Table 4. Finally, gender, working position, and years of experience were not associated with any of the outcomes, and insomnia was not associated with any of the factors included in multivariable logistic regression.

**TABLE 4:** Adjusted* significant risk factors for significant burnout and severe emotional distress.

**Measurement**	**N of severe cases/N total for category (%)**	**OR (95% CI)**	**Category *p-*value**	**Overall *p-*value**
**PFI—significant burnout**				
**Increased workload**				
No	181/433(41.8)	Reference	NA	0.023
Yes	114/214(53.3)	1.48(1.06−2.08)	0.023
**Social support**				
Good	58/149(38.9)	Reference	NA	0.015
Average	192/430(44.7)	1.39(0.94−2.07)	0.101
Poor	45/68(66.2)	2.26(1.24−4.13)	0.008
**DASS-21–severe depression**				
**Social support**				
Good	6/149(4.0)	Reference	NA	<0.001
Average	46/430(10.7)	2.98(1.22−7.24)	0.016
Poor	15/68(22.1)	7.28(2.62−20.23)	< 0.001
**DASS-21–severe anxiety**				
**Occupation**				
Physician	20/179(11.2)	Reference	NA	0.007
Nurse	109/468(23.3)	2.14(1.23−3.73)	0.007
**Age**				
>50 years	19/162(11.7)	Reference	NA	0.009
41-50 years	31/157(19.7)	2.15(1.12−4.13)	0.021
31-40 years	43/179(24.0)	3.28(1.49−7.19)	0.003
26-30 years	22/108(20.4)	2.92(0.95−9.00)	0.062
20-25 years	14/41(34.1)	5.59(1.48−21.21)	0.011
**DASS-2—severe stress**				
**Increased workload**				
No	35/433(8.1)	Reference	NA	0.001
Yes	36/214(16.8)	2.46(1.46−−4.14)	0.001

PFI, 16-item Stanford Professional Fulfillment Index; ISI, 7-item Insomnia Severity Index; DASS-21, 21-item Depression, Anxiety, and Stress Scale. *For job status (e.g., full time, half time, …), marital status, and last 24-h workload.

## Discussion

This mixed-mode cross-sectional survey aimed to evaluate differences in burnout and mental health outcomes among COVID-19 healthcare workers and non-COVID-19 healthcare workers. First, results revealed a high prevalence of burnout, insomnia, depression, and anxiety and a low prevalence of professional fulfillment among participants. Overall, 68.7, 53.3, 52.2, and 40.3% of all participants presented mild to extremely severe symptoms of insomnia, depression, anxiety, and stress, respectively. More precisely, 6% of all participants presented severe symptoms of insomnia, and 10.4, 19.9, and 11% of all participants presented severe to extremely severe symptoms of depression, anxiety, and stress, respectively. Moreover, nurses had more severe symptoms for anxiety than physicians did. Nurses as a group presented a moderate level of anxiety compared to physicians who presented a normal level of anxiety. These results are consistent with those of a recent study that reported high prevalence of mental health symptoms among healthcare workers in China, particularly in nurses (Lai et al., 2020). However, in our study, physicians and nurses did not differ in severity of symptoms for depression and stress. Interestingly, the prevalence of insomnia was considerably higher in our sample, with 68.7% of healthcare workers reporting symptoms, compared to 30.4% in China, and up to 32.7% being above the clinical threshold on the insomnia measurement. In addition, insomnia in CCU was more severe than in NCCU and CCU + NCCU. Measures also revealed a high prevalence of burnout (45.6%) and a low prevalence of professional fulfillment (30.4%) among participants. Prevalence of burnout (overall, 45.6%) was higher than in a previous study (Wu Y. et al., 2020) and particularly for COVID-19 workers (50.0%) but also much higher than the burnout ratio (6%) among Belgian physicians and nurses, reported in a previous national survey (Vandenbroeck et al., 2017).

Second, after adjusting for cofounders, multivariate analysis showed that there was no significant effect of working position on the outcomes. Contrary to our hypothesis, this finding means that healthcare workers’ mental health outcomes did not seem to be associated with the fact to directly treating and caring for patients diagnosed with COVID-19. This is contrary to results of previous studies showing worse mental health outcomes among frontline healthcare workers during the COVID-19 (Lai et al., 2020; Lu et al., 2020; Wu Y. et al., 2020) or H1N1 outbreaks (Matsuishi et al., 2012). However, a significant effect for occupation was found, highlighting worse mental health outcomes among nurses, specifically for burnout, insomnia, and anxiety. In addition, no group × occupation interaction was found. These important findings highlight that, during the COVID-19 outbreak, being a nurse or a physician had a greater influence on burnout, insomnia, and anxiety than working in a COVID-19 or a non-COVID-19 medical care unit or both, at least in our population. First of all, this result may be explained by the fact that in general hospitals, even healthcare workers in NCCU are confronted with the COVID-19, either directly or indirectly. A significant amount of hospitalized patients in NCCU were suspected of having COVID-19. In case of suspected patients, hygiene and protection procedures also applied in these units, but probably with a lack of clear instructions and limited access to personal protection equipment compared to the CCU. Increase in workload, fear of contagion, and risk of infection to their families resulting from unprotected contact with potential COVID-19 patients may lead to the development of anxiety, mental burden, and professional exhaustion for these particular workers just the same as workers in CCU. Consequently, the mental burden of healthcare workers in NCCU may increase if they develop feelings of being inadequately supported or even neglected, as a result of the greater focus on workers in CCU, through widespread media coverage, solidarity actions from the general population, or institutional decisions.

Third, statistical analysis also showed that being a nurse, young, with a poor perceived social support, and an increased workload were risk factors for several outcomes of interest. Thus, nurses were just over two times more likely to have severe symptoms of anxiety and young participants, aged 20–25 years, were almost six times more likely to also have severe symptoms of anxiety. Nurses and younger workers, therefore, appear to be more vulnerable to work- and COVID-19-related stressors. Greater frequency of exposure to the virus for nurses, due to their clinical duties compared to physicians, and for younger workers, due probably to their role in protecting older workers, may lead them to be more exposed to chronic stressors. Interestingly, the level of experience was not reported as a significant risk factor, indicating that work experience does not protect nor does it increase vulnerability to COVID-19 stressors. Poor perceived social support was an independent risk factor for burnout and for depression, leading participants to be seven times more likely to have severe symptoms. It should be noted that the period in which this survey was conducted corresponds to the period of national lockdown aimed to slow down the spread of the virus. This context of social isolation and distancing has arguably complicated the ability for workers to adjust psychologically to an intrinsically challenging management of an acute infectious disease. Increased workload was also reported as a risk factor for severe stress and, as expected, for burnout (Linzer et al., 2001).

Finally, our findings suggest that specific psychological assistance services dedicated to all healthcare workers have to be deployed, given the high prevalence of worse mental health outcomes. This kind of support needs to provide standardized interventions such as psychoeducational sessions about common psychological reactions among nurses and physicians and associated risk factors for burnout, insomnia, and emotional distress, and particularly for young nurses. In the future, healthcare politics and institutions also need to give special attention to the welfare of all the workers by providing systemic response to the specific works environment concerns such as excessive workload, imbalance between clinical demands and workers’ mastered skills, or low staff numbers.

### Study Limitations

This study has several limitations. First, a cross-sectional design provides no information on the evolution over time in outcomes. Second, this study was unable to distinguish the effect of the work environment on emotional distress, and insomnia vs. the effect of the COVID-19 outbreak in general. A comparison with the general population is needed to highlight a specific effect. Moreover, social support, as an independent risk factor, was assessed with a non-validated tool. Third, although the response rate was acceptable, bias in results may still remain if prevalence and severity of mental health outcomes among non-respondents were not similar to those among respondents. Unfortunately, no information was available on non-respondents. However, professional data showed that 20% of respondents, both CCU and NCCU, came from intensive care units. Other hospital specialties may therefore have been underrepresented. Fourth, despite the fact that participants came from five different hospitals, further studies on larger populations are necessary to generalize these kind of results.

## Conclusion

Healthcare workers reported high prevalence of burnout, insomnia depression, and anxiety. However, directly caring for and treating patients diagnosed with COVID-19 is not associated with worse mental health outcomes among healthcare workers. Being a physician or a nurse seems to have a greater influence on mental health outcomes with higher level of burnout, insomnia, and anxiety among nurses. Protecting all healthcare workers during the COVID-19 outbreak needs to be a priority for healthcare institutions and policy makers in order to empower individual and organizational resilience. Given the poor mental well-being among healthcare workers, and specifically among young nurses, their condition needs to be further studied and monitored, and specific interventions need to be rapidly implemented.

## Data Availability Statement

The raw data supporting the conclusions of this article will be made available by the authors, without undue reservation.

## Ethics Statement

The studies involving human participants were reviewed and approved by the Ethics Committee of Erasme Hospital Ethics Committee of Jolimont-Lobbes Hospital. The patients/participants provided their written informed consent to participate in this study.

## Author Contributions

All authors have contributed to the present study and article. JT was involved in conceptualization, data curation, formal analysis, investigation, methodology, project administration, resources, visualization, writing – original draft, and writing – review and editing. MG was involved in conceptualization, methodology, writing – original draft, and writing – review and editing. AL was involved in data curation and resources. AM was involved in conceptualization. J-MH was involved in resources, writing – original draft, and writing – review and editing. GL was involved in writing – original draft and writing – review and editing. MR was involved in conceptualization, methodology, supervision, writing – original draft, and writing – review and editing.

## Conflict of Interest

The authors declare that the research was conducted in the absence of any commercial or financial relationships that could be construed as a potential conflict of interest.
